# Exploring the Multifaceted Potential of Endangered Sturgeon: Caviar, Meat and By-Product Benefits

**DOI:** 10.3390/ani14162425

**Published:** 2024-08-21

**Authors:** Andreea (Stroe) Dudu, Sergiu Emil Georgescu

**Affiliations:** Department of Biochemistry and Molecular Biology, Faculty of Biology, University of Bucharest, Splaiul Independentei 91-95, 050095 Bucharest, Romania; angelica-andreea.stroe@bio.unibuc.ro

**Keywords:** sturgeon aquaculture, caviar, meat, by-products, nutrients, bioactive molecules, biological activities

## Abstract

**Simple Summary:**

Sturgeons are among the most endangered groups of species due to the decline of wild populations, which are threatened by overfishing, habitat loss and pollution. Sturgeon aquaculture involves the farming of sturgeon species primarily to produce caviar but also for their meat and various by-products, which are highly valuable, making sturgeon aquaculture economically and ecologically significant. This sector plays a critical role in reducing the pressure on wild populations by providing a sustainable source of caviar and other sturgeon products. The most renowned product, caviar, has a unique, rich flavor and high nutritional value, including proteins and omega-3 fatty acids. Sturgeon meat is valued for its mild taste and firm texture, also being a nutritious source of protein and essential fatty acids, contributing to a balanced diet. By-products from sturgeon farming, such as cartilage, skin, swim bladders, etc., have the potential to be utilized in various industries. The aim of our research is to explore the multifaceted value of sturgeons, with an emphasis on caviar and by-products. Overall, the efficient use of all sturgeon resources supports the sustainability and profitability of sturgeon aquaculture, contributes to environmental conservation and helps meet the demand for diverse and valuable products.

**Abstract:**

Sturgeons are facing critical endangerment due to overfishing, habitat destruction, pollution and climate change. Their roe, highly prized as caviar, has driven the overexploitation, severely depleting wild populations. In recent years sturgeon aquaculture has experienced significant growth, primarily aimed at providing high-quality caviar and secondarily meat. This sector generates significant quantities of by-products, which are mainly treated as waste, being mostly discarded, impacting the environment, even though they are a source of bioactive molecules and potential applications in various sectors. This article presents a review of the proximate composition and nutritional value of sturgeon caviar and meat, also exploring the potential of the by-products, with an emphasis on the processing of these components, the chemical composition and the functional and bioactive properties. Although sturgeon caviar, meat, and by-products are highly valuable both nutritionally and economically, adopting sustainable practices and innovative approaches is crucial to ensuring the industry’s future growth and maintaining ecological balance. Despite some limitations, like the deficient standardization of the methods for extracting and processing, sturgeon by-products have a tremendous potential to increase the overall value of sturgeon aquaculture and to promote a zero-waste approach, contributing to achieving the Sustainable Development Goals adopted by all United Nations Member States in 2015.

## 1. Introduction

**Figure 1:**
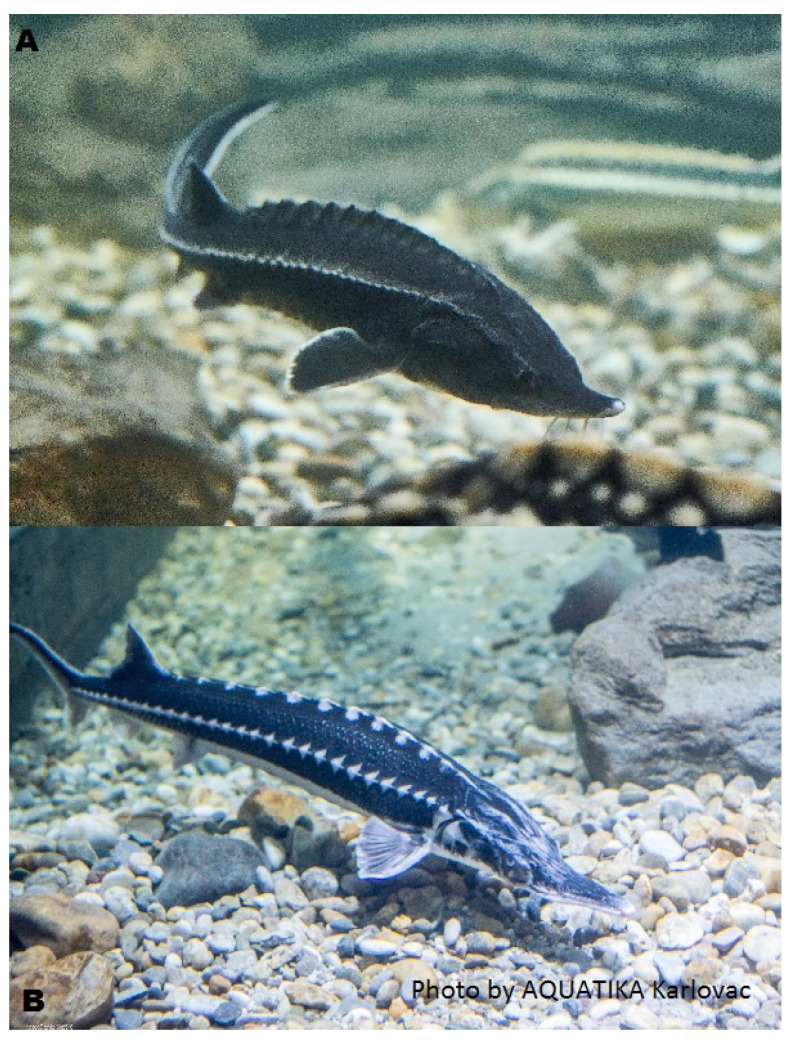
Beluga sturgeon *Huso huso* (**A**) and stellate sturgeon *Acipenser stellatus* (**B**). These species native to the Ponto–Caspian region have distinctive external appearance and traits. Source: FishBase [3,4].

Sturgeons are a group of fish belonging to the family *Acipenseridae*, one of two living families of the Acipenseriformes, alongside paddlefish (*Polyodontidae*). The family is grouped into four genera: *Acipenser*, *Huso*, *Scaphirhynchus* and *Pseudoscaphirhynchus*. Nowadays, there are 25 sturgeon species and two paddlefish species living in fresh and saltwater, native to the Northern Hemisphere, distributed across North America, Europe, and Asia, inhabiting rivers, lakes, and coastal areas. They are known for their ancient lineage, with fossil records dating back over 200 million years. Sturgeons display certain relict features that set them apart from other fish, indicating their ancient origins. Their skeleton is primarily cartilaginous, with partial ossification in only the skull and maxilla. They have ganoid scales only on the caudal part of their bodies, with the rest of their bodies being scaleless but covered with five longitudinal rows of bony plates (Figure 1). Most of the sturgeon species are large, slow-growing fish with late sexual onset, with some species taking 15–20 years to reach sexual maturity in their natural environment. Moreover, they are known for their longevity, with some individuals living for over 100 years [1,2].

Sturgeons are also recognized for their spawning migrations, often traveling long distances between feeding and breeding grounds. Most sturgeons are anadromous species that spend most of their life in salt water and migrate upstream to spawn, while others spend their whole lives in freshwater or migrate between freshwater and saltwater (diadromous species). Certain species have simply transitioned to freshwater habitats, while others were forced by anthropic intervention or by natural changes that have occurred in their native habitats, as is the case with some subpopulations [5].

Currently, most wild sturgeon populations are fragile and need conservation measures. This is the consequence of multiple biological, ecological and economic factors that made this group of fish highly susceptible to overfishing and poaching. The above-mentioned features, like long lifespans, slow growth rates and late sexual maturity, result in low population growth rates and make sturgeons particularly vulnerable to overexploitation. Also, the long intervals between reproductive cycles mean that populations recover slowly from depletion. Anthropic activities such as damming, dredging and river channelization disrupt sturgeon habitats, particularly spawning and juvenile rearing areas. These disruptions reduce the available habitat and environmental aspects necessary for successful reproduction and survival, exacerbating the effects of overfishing and poaching [6]. However, the primary reason for sturgeon overfishing and poaching is the high market value of caviar, which is the unfertilized roe of female sturgeons. This high economic motivation leads to both legal and illegal fishing, despite bans and regulations, and makes sturgeons the world’s most endangered group of species according to the International Union for Conservation of Nature (IUCN) [7]. Sturgeons are listed under Annexes II and I of the Convention on International Trade in Endangered Species (CITES) regulations and are protected all over the world. Many EU countries have banned sturgeon fishing to conserve endangered species. There are legal fisheries for limited quantities in only some countries (e.g., Russia, Canada, USA). Severe depletion of sturgeon stocks has led to the development and expansion of sturgeon aquaculture as a means to meet market demand while conserving wild populations. In the late 1990s, sturgeon farming began to replace traditional fisheries, and farmed caviar has gradually gained market acceptance, becoming a substitute for wild caviar. The global production of sturgeon caviar is currently dominated by aquaculture. This shift is largely driven by the need to protect endangered sturgeon species and ensure a stable supply of caviar without further harming wild populations [8].

Historically the largest caviar producers were Russia and Iran, particularly around the Caspian Sea. Nowadays, the main producer countries of sturgeon caviar from aquaculture include China, Italy, France, the United States and Iran. China is the first sturgeon producer country and the main exporter of sturgeons. In the last decade, the aquaculture of sturgeon has rapidly grown, supported by scientific research focused on breeding, genetic resource management and disease management to optimize production and ensure sustainability [9,10].

The increased interest in sturgeon breeding is also reflected in the number of studies dedicated to this topic. A Google Scholar search for the term “sturgeon aquaculture” generates 2100 results from 2000 until present. The studies follow various aspects, from rearing conditions (temperature, diet, oxygen concentration, disease prevention, etc.) to genetic characterization of stocks, genome manipulation and sustainable sturgeon farming [11].

We can strongly affirm that sturgeon farming has matured over several decades, contributing to both market demands and conservation efforts. Sturgeon aquaculture has become a fast-developing industry with high-level structured businesses supporting commercial farming, processing and trade. Future progress in research depends on improved management and development of sustainable practices. This can be achieved through several key practices and principles like the selection of low-impact aquaculture system, selective breeding and genetic management, sustainable feed practices, efficient resource use, environmental monitoring, conservation programs and valorization of co-products and by-products [12,13].

As a result, advancements in the aquaculture industry have greatly enhanced the role of hatcheries in conserving wild populations. Additionally, these improvements provide a sustainable source of fish for human consumption, thereby reducing the pressure on declining wild stocks [14].

Traditionally, caviar has been the most valued product resulting from sturgeon, yielding substantial commercial value and symbolizing luxury. However, in recent years, the focus of sturgeon aquaculture has expanded beyond this exclusive delicacy. Recognizing the potential of other parts of the fish, the industry has increasingly oriented itself toward valorizing by-products. In this paper, we will use the terms “co-product” with reference to sturgeon meat and “by-products” to refer to all the raw material, edible or inedible, left over following the preparation of the main product [15], like products deriving from sturgeon processing, such as caviar extraction and meat filleting.

This shift from caviar-only production to by-products valorization is driven by a desire to maximize resource efficiency and sustainability, resulting in the development of a wide range of valuable products.

Valorization of sturgeon by-products can greatly influence the sturgeon aquaculture industry in various ways: (i) economic benefits—using all the parts of the sturgeon (like skin, organs, bones, cartilage, etc.) can create additional income streams and can help market diversification; (ii) sustainability and efficient use of resources by waste reduction and the consequent decrease of the environmental footprint of sturgeon farming; (iii) innovation and development of new high-value products (e.g., pharmaceuticals and nutraceuticals) derived from sturgeon by-products, enhancing the overall efficiency and sustainability of aquaculture operations; and (iv) ethical considerations since utilizing the whole fish demonstrates a commitment to ethical farming practices, which can improve the industry’s public image and consumer trust [10,16]. Overall, valorizing sturgeon by-products can enhance the economic viability, sustainability, and innovation within the sturgeon aquaculture industry while supporting conservation and ethical practices.

Aligned with these objectives, this review aims to explore the multifaceted value of sturgeons, with an emphasis on caviar, meat and by-products in various aspects, such as biochemical methods of isolation and purification, nutritional profile, health impacts and applications.

## 2. From Roe to Caviar—Nutritional Profile and Preservation

While fish eggs are commonly referred to as roe, the processed fish eggs are frequently known as “caviar”. Defined as the salt-cured and preserved eggs of fish that have been separated from the supporting connective tissue, sturgeon caviar is not only a gourmet delicacy but also a product with great economic, ecological, and cultural importance. The term “caviar” is only applied to the product coming from sturgeons, which is considered the genuine caviar, while the processed eggs from other species should be labeled accordingly [17].

The value of sturgeon caviar extends beyond its gastronomic appeal. It encompasses significant economic benefits, ecological sustainability, and cultural heritage, making it a unique and impactful product in the global market. Commercially, it represents a high-value product in the luxury food market, having prices that can exceed thousands of dollars per kilogram depending on the variety and quality. 

The selection of species to be raised in aquaculture for caviar production is determined by several factors, like the time needed to reach sexual maturity, which is strongly related to the length of farming before the eggs can be harvested or the biotechnological challenges for rearing. 

In 2016, the most significant sturgeon species farmed for caviar production was *Acipenser baerii* (31% of production), followed by *Acipenser gueldenstaedtii*, the hybrid *Huso dauricus* × *Acipenser schrenckii*, *Acipenser transmontanus* and *Acipenser ruthenus*. Other species and hybrids collectively contributed 45% of the overall caviar yield [8]. According to the CITES Trade database, the caviar traded in 2022–2023 came dominantly from *A. baerii* and *A. gueldenstaedtii*. Besides these two species, other sturgeon species like *A. naccarii*, *A. ruthenus*, *A. persicus*, *A. schrenckii*, *A. stellatus*, *A. transmontanus*, *A. nudiventris*, *H. dauricus* and *H. huso* contributed to the global traded production of caviar. It is also worth noting the rearing of a variety of interspecific hybrids for caviar production [18]. 

The value and cost of caviar are influenced by factors such as the rarity, flavor, texture, and the size of the eggs. Thus, the most valuable and expensive types of caviar are primarily from three species: beluga (*H. huso*), osetra (*A. gueldenstaedtii*) and sevruga (*A. stellatus*) [19].

Beluga caviar is considered the highpoint of luxury and is the most expensive type of caviar available, with retail prices reaching up to USD 10,000 per kilogram [20]. The rarity and long maturation period of the beluga sturgeon females (*H. huso*) (typically, 10–12 years in aquaculture and 14–20 years in the wild) contributes significantly to the high cost of its caviar. The eggs are typically large, with a diameter of 3–4 mm and have a light to dark gray color, a delicate texture and unique, buttery flavor.

Osetra caviar comes from *A. gueldenstaedtii*, a medium-sized species that matures faster than beluga (8–10 years in aquaculture and 10–16 years in the wild), making their caviar more available, but still rare and valuable. The eggs, with a firm texture and nutty flavor, are typically medium-sized, ranging from 2 to 3 mm in diameter and can vary in color from golden yellow to dark brown. 

Sevruga caviar is produced by the stellate sturgeon, which is smaller and matures faster (6–8 years in aquaculture and 10–14 years in the wild) than both beluga and Russian sturgeon. The eggs are the smallest among the three types, typically about 1 to 2 mm in diameter, and are usually dark gray or black [17].

In addition to beluga, osetra and sevruga caviar, there are several other types of sturgeon caviar that are popular among consumers. For instance, the caviar of Siberian sturgeon (*A. baerii*) is known for its small to medium-sized eggs and for its distinctive, slightly briny flavor. The white sturgeon (*A. transmontanus*) caviar is characterized by large, firm eggs with a delicate nutty flavor. It is often compared to osetra caviar in terms of the quality and flavor profile. Kaluga sturgeon (*H. dauricus*) caviar is known for its large, firm eggs, considered an affordable alternative to beluga caviar. Although less common, sterlet (*A. ruthenus*) caviar is appreciated for its fine eggs and mild, buttery flavor. Some caviar comes from hybrid sturgeon species, which combine the desirable qualities of their parent species and can offer unique flavor profiles and textures [21,22,23,24,25].

### 2.1. Nutritional Profile of Sturgeon Roe

Sturgeon caviar is not only prized for its flavor and texture but also for its remarkable nutritional profile. Caviar is a rich source of proteins, lipids, vitamins and minerals, making it a nutrient-dense food that offers several health benefits. The composition of sturgeon roe, as well as that of other fish species, depends on various factors, including the species, diet, environment, aquaculture practices and method of processing [26,27]. It is considered a high-quality food with important amounts of protein and amino acids. Sturgeon caviar contains lipids important for maintaining human health. It is known that sturgeon is rich in DHA—docosahexaenoic acid (C22:6n-3)—and EPA—eicosapentaenoic acid (C20:5n-3)—which play an important role in the prevention and treatment of cardiovascular diseases and are also called “brain gold” since they have proven to improve IQ and prevent senile dementia [28,29,30].

Table 1 reports detailed information on several key features of caviar, including the water, protein, and lipid content from different sturgeon species. These parameters are crucial for understanding the quality and sensory characteristics of caviar.

#### 2.1.1. Water Content

The water content affects the texture and shelf life, with higher moisture levels potentially leading to a softer texture and shorter shelf life. Several studies have demonstrated that the water content found in the eggs of different fish species is very variable, ranging from 45 to 90% [30]. In sturgeons, the proximate water content of fresh roe varies from 45% to 65% depending on the species. In processed caviar, the water content depends on the salt content. 

#### 2.1.2. Protein Content

Generally, fish roe contains approximately 75% ovoglobulins, 13% collagen, and 11% albumin. The protein content is essential for evaluating the nutritional value and functional properties of caviar, such as its ability to form gels or emulsify. In most cases, the protein content ranges from ~15 to 31%, proving that sturgeon caviar is a rich protein source, with high nutritive value. The most valuable caviars, e.g., beluga, osetra, sevruga and imperial, show a protein content of ~24–31%, comparable to that of salmon and mullet roes [39].

#### 2.1.3. Lipid Content

Most sturgeon roe is quite fatty, containing 10–20% lipids, which contributes to the rich, melting texture of the caviar, which is one of its most prized sensory qualities. This fat content is comparable with that found in capelin (*Mallotus vilosus*) (18–19%), mullet (*Mugil cephalus*) (13.7%) and chum salmon (*Onchorhynchus keta*) (13.5%) [17].

#### 2.1.4. Carbohydrate Content

The carbohydrate content of sturgeon roe is generally minimal, which may explain why it has not been extensively measured. The limited data presented in Table 1 show the carbohydrate content of three types of caviar, ranging from 4.60 to 6.90 g/100 g wet weight.

#### 2.1.5. Amino Acid Composition

Different studies reported variable amino acid composition depending on the species, diet, habitat and age. Sturgeon caviar is an important source of both essential amino acids (EAA) and non-essential amino acids (NEAA). The analysis of the amino acids profile has demonstrated that the caviar is well balanced and complete, providing all the essential amino acids that the human body cannot synthetize on its own. A study by Gong et al., 2013 [32], showed that the content of EAA is lower than the content of NEAA in caviar samples from farmed Siberian sturgeon, Amur sturgeon and a sturgeon hybrid. For these species, the most abundant amino acids is glutamic acids (7.29–7.69%), followed by aspartic acids, leucine, lysine, serine and arginine. The content of isoleucine, leucine, lysine, valine, glycine and tryptophan is significantly higher in Siberian sturgeon compared with the sturgeon hybrid.

The EAA content is comparable to other fish roes like salmon and cod, but differences were reported concerning the specific amino acid composition and ratios. Moreover, the total EAA content in sturgeon caviar, as well as the concentration of each EAA in the three types of caviar, is higher than the reference FAO/WHO pattern [32]. For caviar obtained from beluga, Russian, and Persian sturgeon, aspartic acid is the most abundant amino acid, followed by glutamic acid, lysine and serine. The ratio EAA/NEAA ranges from 0.99 to 1.23, making caviar a source of high-quality protein [34]. Ovissipour and Rasco (2011) showed that glutamic acid and aspartic acid are the amino acids with the highest concentration in roes from farmed and wild beluga, followed by lysine, arginine, valine and isoleucine [36]. Hamzeh et al., 2015 [37], found that the glutamine, serine, alanine, methionine and lysine content is significantly higher in wild beluga caviar than in farmed beluga caviar. Other studies comparing the amino acids content of wild and farmed beluga caviar demonstrated that there is no significant difference in the total content of EAA and NEAA between wild and farmed caviar [36,37,38].

#### 2.1.6. Fatty Acids Profile

The lipids in roes primarily consist of triacylglycerols (TAGs), phospholipids (PLs), waxes, and cholesterol. While no significant qualitative differences in the fatty acid (FA) profiles of various fish roes were identified, quantitative variations in their levels were apparent. The FA profile in TAGs is influenced by the fish diet, while the FA patterns in PLs are influenced by the water temperature, in addition to the food composition. During gonadogenesis, SFA and MUFA from the female sturgeon lipid reserves are primarily catabolized to supply energy for egg synthesis. In contrast, particularly long-chain n-3 fatty acids (PUFA) accumulate in the egg lipids. Consequently, the fatty acid composition in different lipid fractions of eggs varies significantly and should be assessed separately due to their distinct biological functions [24]. In this light, the FA profiles from PLs and neutral lipids (NLs) seem to be more informative for assessing the quality of caviar. The analysis of caviar in different sturgeon species showed a prevalence of unsaturated fatty acids with respect to saturated fatty acids (SFAs), with the polyunsaturated fatty acids (PUFAs) content being between 40% and 50% of the total FA when compared with monounsaturated fatty acids (MUFAs) [31].

The analysis of raw eggs and caviar in three farmed sturgeon species (Siberian sturgeon, Russian sturgeon and white sturgeon) showed that the main MUFA was represented by oleic acid (18:1 n9), with white sturgeon caviar showing the highest value (30.6%), followed by Siberian sturgeon (28.4%) and then Russian sturgeon (25.4%) caviar. There is a consensus in the scientific literature that oleic acid is the most abundant fatty acid in sturgeon eggs, irrespective of their origin and species [31,35,40,41,42,43]. Linoleic acid (LA, 18:2 n-6), arachidonic acid (ARA, 20:4 n-6) decosahexaenoic acid (DHA, 22:6 n-3) and eicosapentaenoic acid (EPA, 20:5 n-3) are the main PUFAs in caviar found in different proportions depending on the species and origin. Linoleic acid has been used to distinguish the origin of eggs from farmed and wild sturgeons because of its higher content in caviar from cultured sturgeon, mainly due to the lipid composition of commercial diets rich in oils of vegetable origin (soy oil, sunflower oil, rapeseed oil or corn oil) [31,40,41,44,45]. Similarly to LA, the level of ARA in caviar is influenced by the feed given to sturgeons. ARA is abundant in natural aquatic food chains, especially in certain algae and crustaceans, which are typical components of wild sturgeon diet, and for this reason is found in a higher amount in wild caviar compared to framed caviar [46]. Among the n-3 PUFA series, DHA and EPA are the most abundant. Apparently, the deposition of these FAs in sturgeon eggs is not dependent on the type of the diet. Both wild and farmed sturgeon caviar have shown similar levels of EPA and DHA, indicating that diet composition has a minimal effect on the accumulation of these fatty acids in fish eggs. Moreover, similar concentrations of these fatty acids in caviar from sturgeons fed diets with either squid oil or soybean oil as the primary lipid source were reported in white sturgeon [40]. 

The proportions of n-3 and n-6 FAs in the diet are crucial, especially for neural function. Achieving an ideal ratio of 2:1 or 1:1 is important for neurodevelopment and can help prevent early neurodegeneration [47]. The n-3/n-6 ratio varied from 0.9 to 1.40 in both raw eggs and caviar from Siberian sturgeon, Russian sturgeon and white sturgeon raised in aquaculture. The same value was obtained in a study regarding the quality of farmed caviar from Amur sturgeon, Siberian sturgeon and a hybrid. However, this ratio is lower in sturgeon caviar compared to that reported for other marine fish eggs [31,32].

A study by Ovissipour and Rasco, 2011 [36], demonstrated that the wild beluga roe contains about twice the total n-3 fatty acids compared to farmed beluga roe. Instead, farmed beluga roe has higher n-6 levels, with linoleic and linolenic acids being the most abundant. The higher n-3/n-6 ratio in wild beluga suggests better health and energy reserves, while a low ratio negatively affects fertilization and hatching rates. Enhancing n-3 in the diet of farmed beluga is recommended for better roe quality and reproductive success [31]. Overall, when compared to wild sturgeon caviar, farmed sturgeon caviar has a lower lipid content and less arachidonic acid, but a higher n-6 PUFA level, with linoleic acid being predominant among these types of FAs. Instead, wild caviar has a higher amino acids content, more oleic acid and more n-3 PUFA compared to the farmed caviar [39].

The development of sturgeon aquaculture has also been facilitated by high-energy diets designed to accelerate growth. Research continues to refine these diets to balance growth rates with optimal reproductive health and caviar value. Overall, the farmed caviar quality can be very high due to controlled breeding, feeding and harvesting practices—sturgeon farming can, thus, help alleviate pressure on wild populations by providing an alternative source of caviar.

#### 2.1.7. Minerals and Vitamins

Beside essential amino acids and high-quality PUFAs, sturgeon roe and caviar contain a variety of essential minerals and vitamins that contribute to their nutritional value. The key minerals found in sturgeon caviar include sodium, phosphorus, calcium, magnesium, potassium, and iron. For example, sturgeon roe from *H. huso*, *A. stellatus*, *A. gueldenstaedtii*, and *A. baerii* were found to contain varying levels of minerals, including zinc (10.3–12.4 mg/kg), copper (1.20–1.69 mg/kg), and lead (0.06–0.15 mg/kg) (wet weight) [26]. 

DePeters et al. (2013) [43] investigated the mineral composition of caviar from *A. transmontanus* to determine if it could be used to distinguish between farmed and wild sturgeon. The concentrations of several minerals (reported as mg/kg dry weight), like iron (67.3–70.6), zinc (54.6–59.4), copper (8.3–9.4), phosphorus (9676–9716), sulfur (6706–6945), calcium (311–313), and potassium (3578–4078), did not differ between farm-raised and wild eggs. However, they observed higher levels of Mn, Mg, Na, Se, and As, along with lower levels of Ba, in wild sturgeon eggs compared to those from farmed sturgeon [43]. Regarding vitamins, sturgeon caviar is a rich source of several B vitamins, particularly B12. For example, white sturgeon (*A. transmontanus*) caviar contains substantial levels of B12 (~15 µg/100 g wet weight). It also contains significant amounts of vitamins A, E, and D [48].

When compared to other fish roe, sturgeon caviar generally has higher levels of certain vitamins and minerals. For instance, it often contains more vitamin B12 than roe from species like salmon and trout. However, the exact concentrations can vary based on the species of sturgeon, their diet, and whether they are wild or farmed. Overall, sturgeon caviar is a highly nutritious food that stands out for its rich content of essential minerals and vitamins, particularly phosphorus, calcium, and vitamin B12, making it a superior choice compared with many other types of fish roes [39,48].

### 2.2. Raw Eggs versus Caviar—Nutritional Status

The nutritional composition of raw sturgeon eggs differs from that of sturgeon caviar due to the processing methods involved in creating caviar. The quality of caviar is primarily determined by the quality of its raw material. Alteration can occur due to physical, chemical and microbiological changes that happen during the processing of raw eggs and caviar storage, leading to differences in product quality.

Sturgeon eggs are harvested for caviar production from mature females through various methods, including traditional or modern approaches. The traditional method involves killing the female sturgeon to extract the eggs. Modern methods allow the extraction of eggs without killing the fish. For example, such a method involves using ultrasound to determine the right time for harvesting, administering a protein to release the eggs into the body cavity, and then massaging the eggs out of the fish. This method allows for multiple harvests over the female sturgeon’s lifetime [49].

After extraction, the eggs are carefully removed, chilled and the ovary membranes are carefully removed. The eggs are then repeatedly washed in cold water to remove impurities, any damaged eggs, and membrane debris. Next, the caviar is sorted, weighed and salted. The salt content varies from less than 3–10%, with high-quality caviar typically containing less than 3% salt. The caviar is packed in small tins, which are hand-filled and gently pressed to remove air, then tightly sealed to prevent oxidation. Caviar is then aged for three months to develop its flavor, with fresh caviar typically being storable for 2–4 weeks. Freezing, drying and pasteurization can extend the shelf life, with pasteurized caviar lasting up to one year at room temperature [50]. Mild temperatures (50–70 °C) are used during pasteurization to account for the thermal sensitivity of roe and to maintain its physical appearance without causing protein coagulation, thus preserving the quality of the proteins. Excessive salting disrupts the taste and appearance of caviar, also leading to egg dehydration. Caviar produced using the dry method and below 4% salt at ambient temperature was found to be higher in water content (52–56%) than caviar produced using brine solution at 70 °C (31.9%) [39]. Salting reduced the moisture content of raw roe by lowering the water activity while simultaneously increasing the mineral content. For example, a high salt content (8%) leads to increased dehydration of the egg and to lower water content (37–42%) [26].

Both raw eggs and processed caviar contain essential nutrients, but the specifics of these nutrients are variable. As previous mentioned, the raw sturgeon eggs are packed with high-quality proteins, essential amino acids and a significant amount of omega-3 and omega-6 fatty acids. They also contain vitamins such as A, D, and B12, as well as minerals like phosphorus, selenium, magnesium, and zinc. The nutrient composition is particularly geared toward the reproductive needs of the fish, providing a dense source of energy and essential nutrients.

Sturgeon caviar, made from processed and salted sturgeon eggs, retains many of the nutritional benefits of raw eggs, but with some differences due to the salting process. Similar to raw eggs, caviar is rich in essential amino acids, PUFA including EPA and DHA, vitamins A, D, E, and B12 (although the levels can be slightly altered due to processing) and minerals. The salting process involved in making caviar can increase the sodium content significantly, which is a major nutritional difference from raw eggs.

Additionally, the lipid profile might see slight changes, but caviar generally retains a high concentration of beneficial fats. During caviar storage, several reactions affecting lipid fractions like the oxidation of PUFA can affect the nutritional properties of caviar. Additionally, enzymes like lipases and phospholipases induce lipolysis, leading to fatty acids that can undergo auto-oxidation. Low-molecular-weight compounds resulting from FA auto-oxidation lead to rancid taste. However, the analysis of caviar from farmed white sturgeon caviar concluded that lipolysis and lipid oxidation are desirable reactions, producing 2,4-alkadienals and n-alkanals, which are the main chemicals responsible for caviar’s flavor [17,39,40,51].

## 3. Sturgeon Meat—Main Product or By-Product?

The classification of sturgeon meat as a main product or a by-product largely depends on the primary focus of the sturgeon farming or fishing operation. In aquaculture operations where caviar is the primary product, sturgeon meat is often considered a by-product. The economic value of caviar significantly surpasses that of meat, leading to operations that prioritize roe extraction. A report by the European Commission established that the world production of caviar was estimated at 340 tons in 2016, with China being the principal producer. In the EU, the caviar production was 126 tons in 2016, while consumption was between 101 and 106 tons. The assessment of consumption is difficult to make since no database exists and this leads to important deviation in the data from different sources. The other main consumers outside the EU are USA, Japan, Russia, China and Australia. The prices are very variable depending on the species from which the caviar was produced, origin and volume. From 2014 to 2017, average EU import price for caviar (mainly of Chinese origin) has dropped from more than 400 EUR/kg to just above 200 EUR/kg, while the average intra-EU export price fell from 428 EUR/kg to 370 EUR/kg and the extra-EU export price decreased from 443 EUR/kg to 396 EUR/kg. In 2017, in the US market, the import prices for caviar varied from 209 EUR/kg to 466 EUR/kg. The highest prices recorded in 2017 were for caviar from Iran, ranging from 1548 EUR/kg to 2237 EUR/kg. Regarding the caviar trade’s predictions for the future, there are increasing concerns that there will be more production than demand, leading to a decrease in prices and profitability [52]. It is important to bear in mind that the import/export price is much lower than the retail price because it does not include the additional costs involved in bringing the finished product to specialized stores. Thus, the retail price of caviar is still very high, in accordance with its reputation as a luxury item.

The economic viability of a sturgeon farm relies on the existence of a meat market, even in aquaculture facilities where sturgeons are primarily reared in aquaculture to produce caviar. It is estimated that around 20 tons of sturgeon meat are produced for each ton of caviar. In 2020, global aquaculture production of sturgeons was 123.476 tons. The major exporters of sturgeon meat are China, Armenia, and Italy. In 2018, these three countries made up 88% of the total sturgeon meat exports. Most countries with high sturgeon meat production also have significant exports of caviar. The exception is Armenia, which has very low levels of caviar exports, mainly because this country has a culture of raising sturgeon primarily for meat consumption [53]. Sturgeon meat is considered a main product in meat-centric or dual-purpose operations. In some regions and operations, sturgeon meat is regarded as a main product. This is particularly true where there is a significant market demand for the meat, which is considered a delicacy.

For example, China is emerging as a significant player in the sturgeon market, not only in terms of caviar production but also in consuming sturgeon meat. In this country, sturgeon farming is still mainly oriented toward meat production. The increasing production capacity in China is meeting both domestic and international demands [8]. 

Sturgeon meat consumption has longstanding traditions and cultural implications in several countries, particularly those around the Caspian Sea and in parts of Eastern Europe and Asia. Russia has a rich history of sturgeon consumption, especially given the country’s access to the Caspian Sea and the Volga River. In Iran, particularly along the Caspian Sea coast, the consumption of sturgeon meat is part of the local culinary tradition. China has a historical tradition of consuming sturgeon, particularly in regions along the Yangtze River where the Chinese sturgeon is found. In this country, sturgeon meat is valued not just for its taste but also for its perceived health benefits. In Eastern European countries, due to the proximity to the Black Sea and Caspian Sea, sturgeon was perceived as a valued food source historically. These regions have developed unique recipes and methods for preparing sturgeon that reflect their cultural identities.

In Western Europe, countries like Italy and France have shown an increasing interest in sturgeon meat. Italy accounts for over half of the sturgeon meat consumption in Europe, with projections indicating a rise in demand. Additionally, in Western France, particularly around Bordeaux, sturgeon meat is popular due to the traditional knowledge and historical presence of sturgeon in the Garonne and Dordogne rivers. Furthermore, countries in Central Europe, like Poland, Germany, Lithuania, Estonia, and Latvia, are involved in sturgeon farming, contributing to both local consumption and exports. These regions recognize the high nutritional value and culinary appeal of sturgeon meat, positioning it as a gourmet product in their markets [49,54,55,56,57]. 

Like caviar, meat from different sturgeon species has different market values. Sturgeon meat is sold fresh, frozen, and smoked as whole fish, steaks, and fillets. The price of sturgeon meat varies depending on the sturgeon species as well as the cut and preparation of the meat. Some producers claim that meat from beluga sturgeons is especially pricey (both fresh and smoked) due to the longer time that is required before this species can be harvested [53]. According to experts’ opinions, the sturgeon meat with the highest quality is obtained from white sturgeon (*A. transmontanus*), although the meat of other sturgeon species and hybrids is also highly appreciated [31]. 

Most stakeholders (producers and distributors) reported selling fresh and frozen sturgeon meat on the EU market for prices of 6.00–8.00 EUR/kg. However, some mentioned that sturgeon meat can fetch up to 50.00 EUR/kg, with male sturgeon meat generally having a higher price than that of females. The price of smoked sturgeon fillets ranges from 75.00 to 120.00 EUR/kg when sold online and in high-end caviar retail stores, where it is marketed as a luxury product. Smoked meat processed into pastes and slices has an average price of 167.00 EUR/kg. Retailers in China mainly sell fresh, small sturgeons of 1–2 kg for meat consumption at an average price of 3.5–4.5 EUR/kg [53].

### 3.1. Nutritional Profile of Sturgeon Meat

To date, the chemical quality of meat from various sturgeon species and hybrids of different ages and weights has been studied. The main conclusions of these studies are that the lipid content of farmed sturgeon meat varies depending on the species and the size of the specimens, with the FA profile of sturgeon meat showing significant amounts of valuable omega-3 fatty acids, particularly EPA and DHA, reflecting the sturgeon diet’s composition of FA. The protein content has been noted for its high biological value, attributed to the amino acid composition.

The proximate composition analysis of sturgeon meat from different species is summarized in Table 2.

For fish that were fed with the same diet and for which the environmental conditions were the same, it is reasonable to suppose that genetic factors form the basis for the differences observed regarding the protein and lipid differences, although an age effect cannot be ruled out [59]. The differences observed regarding the proximate composition in the groups of fish for which different diet formulations were experimented were very variable, from almost no difference to a significant difference, reflecting the importance of the diet and feeding regime in sturgeon aquaculture. Overall, sturgeon meat is a medium-fat high-protein product, making it an appealing food for consumers.

#### 3.1.1. Amino Acids Composition

The amino acids composition of sturgeon meat has been studied and shows a high nutritional value due to the presence of essential and non-essential amino acids. 

The analysis of essential and non-essential amino acids in raw meat of beluga sturgeon (*H. huso*) revealed that the most prevalent amino acids were glutamic acid (18.1%), aspartic acid (16.1%), leucine (9.6%) and lysine (8.5%) [66]. The amino acids composition of the meat of Persian sturgeon (*A. persicus*) was similar to the composition of *H. huso*. The total NEAA (57.85%) content was higher than the EAA content (42.97%). Among them all, glutamic acid showed the highest concentration (18.4 ± 0.06%), followed by aspartic acid (9.94 ± 0.01%), lysine (9.42 ± 0.25%) and leucine (8.45 ± 0.19%) [67]. Kenari et al. (2009) studied the amino acids of cultured beluga of different ages (0.25, 1, 2, 3, 4 and 5 years old). The analysis of *H. huso* fillets indicated that the predominant amino acids were glutamate, lysine, leucine, and aspartate. The percentages of savory-tasting amino acids (aspartate and glutamate) and sweet amino acids (glycine and alanine) relative to the total amino acids were notably high, ranging from 23.9% to 29.2% and 10.8% to 12.0%, respectively. The total content of EAA varied significantly with age, with values between 42.0% and 45.0%. Additionally, the EAA/NEAA ratio ranged from 0.730 to 0.819, higher than the FAO/WHO recommended value of 0.6. The ratio was highest in 5-year-old fish, suggesting that relatively more essential amino acids are produced as the fish age [68]. Badiani et al. (1996) also found that the primary amino acids in raw cultured sturgeon (*Acipenser* spp.) were glutamic acid, aspartic acid, lysine and leucine [69].

Overall, sturgeon meat has a well-balanced content of both EAA and NEAA and a high score of EAA, although ulterior processing like smoking can negatively affect some essential amino acids [66].

#### 3.1.2. Fatty Acids Profile

The specific fatty acids composition (Table 3) can vary depending on factors such as the diet, habitat, and farming practices, highlighting the need for species-specific assessments to optimize their nutritional value and marketability.

The FA composition of sturgeon meat revealed a higher proportion of unsaturated fatty acids compared to saturated fatty acids across in all the studies except one [70]. The total content of MUFA and PUFA were variable depending on species. In some cases, MUFAs were prevalent over PUFAs and vice versa. For example, in the study by Lopez et al. (2020) [31], meat from male white sturgeon showed a higher PUFA content (44.2%) compared to meat from female white sturgeons used for caviar production (33.9%) and lightweight caviar-designated female Siberian sturgeon (35.2%). This finding supports the hypothesis that female sturgeons selectively deposit essential fatty acids (ARA, EPA, DHA) in their eggs for reproduction, while other fatty acids (primarily OA, LA and ALA) accumulate in their fat deposits. The MUFA/PUFA ratio seems to depend on the diet formulation, as is the case for Russian sturgeon juveniles fed with two experimental diets. Fish fed with a diet enriched in canola oil showed higher levels of MUFA (with oleic acid being predominant) and lower levels of n-6 PUFA when compared to the group fed with a diet enriched in sunflower oil. After 15 weeks of rearing, most fatty acids in the muscle of Russian sturgeon reflected the fatty acid profile of the diet [63]. 

The n-3 and n-6 PUFA content is highly variable among species, also reflecting the characteristics of the feeding regime and rearing conditions. The high levels of EPA and DHA in sturgeon meat are in favor of promoting this product on the market as one with a reduced lipid content with high nutritional value. This information could change the existing opinion about sturgeon meat as a by-product of caviar, enhancing the prospects for sturgeon aquaculture focused on meat production [31].

#### 3.1.3. Minerals and Vitamins

Sturgeon meat is rich in essential vitamins and minerals, contributing significantly to a balanced diet. Vitamins D and E are among the most important fat-soluble vitamins found in sturgeon meat. Regarding the water-soluble vitamins, vitamin B6, B12, niacin and pantothenic acid have fairly high content.

Sturgeon flesh is characterized by a low content of sodium, so it could therefore be selected for inclusion in low-sodium diets. This product contains significant amounts of minerals such as phosphorus, selenium, magnesium and potassium. The calcium levels are generally low in sturgeons, as long as the bones are carefully removed. Sturgeon, like most fish, generally does not have high iron content unless their edible portion includes a significant amount of dark muscle [69].

There are some variations in the vitamin and mineral content between different sturgeon species, reflecting the diet, habitat, season, body size and aquaculture practices. However, all the species generally provide a rich source of these nutrients, making sturgeon a valuable addition to a healthy diet.

## 4. Unlocking the Potential of Sturgeon By-Products

While human consumption options for sturgeon products like caviar and meat remain limited, there are many possibilities for value addition. Far from being “waste”, the by-products of these valuable species represent a good source of value-added compounds. Previously, fish by-products were mainly used for making low-value products such as animal feed, silage or fertilizer components. For a large amount of time, there was great ignorance about the effective management of “waste” and the benefits that it provides, leading to its disposal in water bodies with a tremendous negative impact on the environment [73]. However, in the last decade, advancements in processing and the development of new products have been highlighted. There is an actual trend toward utilizing fish by-products in sustainable processes to enhance fisheries’ waste management and mitigate negative impacts.

Consequently, considerable attention has been paid to the nutrients and bioactive compounds present in fish by-products.

These materials are considered sustainable sources for a variety of products and markets, like the pharmaceutical, nutraceutical, food, cosmetic and medical sectors [74,75]. The by-products usually include the head, viscera, skin, bones, and scales, with ranges of 9–12%, 12–18%, 1–3%, 9–15% and 5% of the whole fish weight, respectively [76] (Table 4).

### 4.1. Nutrients and Bioactive Compounds from Sturgeon By-Products

Fish bioactive compounds are constituents found in fish by-products with biological activity. These substances have possible benefits for human health due to their multiple biological activities, such as the antioxidant activity, anti-inflammatory action, hormones mediation, immune function improvement, etc. [74]. Thus, many studies have been directed toward extracting bioactive compounds from different by-products, as will be discussed in the following sections.

#### 4.1.1. Proteins and Active Peptides

As previous shown, both sturgeon caviar and meat are rich sources of protein, containing a well-balanced amount of essential and nonessential amino acids. Beside these, several by-products, such as skin, head, viscera, and bones, are source of protein hydrolysates, bioactive peptides, proteoglycans, collagen/gelatin, etc.

Protein hydrolysates have been isolated from different sources since the modification by enzymes or chemicals improves the functional properties of native proteins and their usefulness as intermediate ingredients in the cosmetics, pharmaceutical, food and nutraceutical sectors [76,77]. To obtain such types of hydrolysates from fish by-products (e.g., viscera), it is necessary to follow several steps: isolation or pretreatments, followed by hydrolysis and protein recovery. Pretreatments comprise a group of processes targeting concentrate proteins at to remove fat and other undesirable components that contribute to the oxidation and coloration of the final product, leading to unpleasant smells and tastes and highly toxic compounds [78]. An alternative to isolate proteins from fish tissues involves the solubilization of protein in acids or alkaline solutions, in addition to centrifugation and filtration to remove insoluble compounds, followed by the proteins’ precipitation by adjusting the pH to the isoelectric point and recovery by centrifugation or decantation [76,79]. 

Protein hydrolysis involves the cleavage of peptide bonds to obtain free amino acids and low-molecular-weight peptides. The hydrolysis process can be chemical or biochemical, with each of these having advantages and limitations. For example, the conventional acid hydrolysis method involving sample treatment in excessively acidic solutions at high temperatures has a low cost, and it is a quick and simple operation, which makes it applicable at the industrial level. However, essential amino acids, such as tryptophan, methionine, cystine and cysteine, are usually destroyed, and asparagine and glutamine are converted into aspartic acid and glutamic acid, respectively. Moreover, the hydrolysates have poor functional characteristics due to the formation of salts after the neutralization process [80]. 

Other variants are represented by the (i) enzymatic hydrolysis process—usually performed in a reactor with temperature, pH, agitation and time controls and (ii) autolysis (biochemical hydrolysis) by using proteases present in the digestive system of fish, such as pepsin, trypsin, chymotrypsin, collagenase and elastase at a controlled pH, temperature and time. The last one, despite the low cost, is difficult to standardize and control because endogenous enzymes depend on several factors, including the seasonality, type and amount of enzymes, fish species and others [81].

The protein recovery process can be realized by different methods, such as centrifugation, nanofiltration, ultrafiltration, microfiltration and ion exchange chromatography, depending on the final use of the hydrolysates [80].

Obtaining protein hydrolysates is usually followed by the separation of different peptide fractions based on the molecular weight, since there is a proven relationship between peptide sizes and certain bioactive, antioxidant and functional properties of hydrolysates [78,82]. Such peptides consist of short amino acid chains that are inactive within the precursor protein and are called functional peptides. These biomolecules have been studied in the last few years due to their antioxidant, antihypertensive, antimicrobial and anti-inflammatory properties [83]. Different fish species by-products are rich in bioactive peptides. Several studies have shown that the head, viscera, skin, and backbone are good sources of protein hydrolysates containing functional peptides. Especially, some collagen-derived peptides could exhibit interesting antioxidant activity, antimicrobial activity against different strains of bacteria and antihypertensive activity through ACE inhibitory properties [84]. 

In sturgeon, several studies have been directed toward optimization of the process to obtain protein hydrolysates and functional peptides and to characterize these biomolecules regarding both the composition and the function. 

Ovissipour et al. (2009) studied the effect of enzymatic hydrolysis and temperature on the protein hydrolysates isolation from Persian sturgeon (*A. persicus*) viscera. The authors concluded that the alcalase treatment can make the recovery rate of sturgeon visceral protein reach 83.64%. The protein hydrolysate was characterized by the following proximate composition: 65.82% protein, 0.18% fat, 4.45% moisture and 7.67% ash, having an important content of EAA and NEAA, with glutamate/glutamine, methionine, aspartate/asparagine and lysine having the higher proportion [85]. The hydrolysis of sturgeon viscera protein using different enzymes and chemical reactions led to hydrolysates with various degree of hydrolysis, protein content and protein recovery. For example, biochemical hydrolysis led to protein hydrolysates with higher protein content [86]. 

#### 4.1.2. Collagen and Gelatin

Collagen is the most abundant single protein present in fish. It is an extracellular fibrous and structural protein with an important role in the physiological function of tissues in bones, tendons, skin, head, cartilage and muscle [87]. Collagen has multiple uses in cosmetics, the pharmaceutical industry and medical care (including esthetic medicine, orthopedics, ophthalmology and dentistry). There are many types of collagen, but the most common form in fish by-products is collagen type I, although from some sturgeon by-products collagen type II was extracted, as will be detailed later in this article. Furthermore, fish collagen, following its extraction, may be further enzymatically hydrolyzed to release physiologically active peptides with important biological activities [88,89].

Gelatin is a macromolecule resulting from the irreversible thermal denaturation of collagen, with which it shares a kinetically similar composition and some of its properties. It is used for improving the consistency, elasticity and stability of foods, as well as to produce edible and biodegradable films that increase the shelf life of food products [87,88].

#### 4.1.3. Lipids

In fish, lipids are found in the subcutaneous tissue, viscera, muscle tissue, liver, mesenteric tissue and head and can be extracted from several by-products like skin, viscera, head and bone from different fish species. Sturgeon oil obtained from by-products, similar to others fish oils, contains valuable PUFA with numerous benefits for human health. Many studies have shown that sturgeon is rich in EPA and DHA, two bioactive substances with anti-inflammatory properties with cardio- and neuroprotective benefits [26,31,34,35,36,40,41,43]. Beside these two n-3 FAs, important amounts of oleic acid, linoleic acid and linolenic acid have been found in different sturgeon by-products. For example, in the skin of a sturgeon hybrid (*A. baerii* × *A. schrenckii*), linolenic acid, DHA and linoleic acid were the most prevalent PUFAs [90].

The FA profile was investigated in different tissue (liver, muscle, gonad, gill, brain, kidney, adipose tissue) of *A. baerii*, showing that these are important sources of highly value FA. The Siberian sturgeon is able to selectively accumulate highly unsaturated fatty acids (HUFAs) in particular tissues, by either the incorporation of these FAs from dietary sources or the desaturation and elongation of PUFAs in its tissues [71].

#### 4.1.4. Minerals

Fish by-products generate a huge amount of minerals, especially the bones, which are an important source of hydroxyapatite, calcium, phosphate, zinc, selenium and iron [91]. These minerals are important compounds in nutraceutical formulations designated to improve health, mainly bone health but also cardiovascular or immunological health [88,92]. 

Different studies have shown that not only caviar and meat are important sources of minerals but also sturgeon bones and cartilage, which contain rich types of trace elements required by the human body. In the cartilage of sturgeon from the Yangtze River Basin, important amounts of iron, cooper, zinc and other trace elements were detected [10]. These trace elements were detected in sturgeon year round, having a periodic profile, with sodium, magnesium, potassium, manganese and iron being the highest in spring, while sodium, calcium and zinc are the highest in summer [93].

Glycosaminoglycans (GAGs) are polysaccharides composed of repetitions of disaccharides (generally formed by a unit of uronic acid and a unit of an amino sugar) linked by an O-glycosidic bond. GAGs like chondroitin sulfate, dermatan sulfate or heparin sulfate are part of the connective tissue, forming the extracellular matrix together with collagen and other structural molecules [94]. These molecules have many biological activities. One of the most significant and also most well known is the anticoagulant activity of heparin.

The GAGs can bind to proteins and form proteoglycans, which can bind growth factors and thus they have great importance in the process of cellular differentiation and function. By these features, GAGs (especially hyaluronic acid and chondroitin sulfate) are particularly appropriate for tissue regeneration and are used in the field of regenerative medicine [95].

In sturgeon, anti-inflammatory and antioxidant GAGs from bones and cartilage have been studied in recent years because they can act as efficient adjuvants, together with other immune factors, to support the adaptive response and promote wound healing. Also, they inhibit the angiotensin-converting enzyme (AGE) and have anticoagulation effects [96]. Chondroitin sulfate isolated from Siberian sturgeon cartilage has an antioxidant effect that increases with the sulfate concentration. Moreover, this GAG has been proven to significantly increase the immune function and anti-inflammatory and anti-allergic activities of mice [10].

### 4.2. Current Progress and Studies on Sturgeon By-Products

#### 4.2.1. Bones and Cartilage

These represent important sources of bioactive molecules with a variety of biological activities. 

The cartilage content is relatively high in sturgeon. The cartilage from the head, notochord and fin represent approximately 10% of the body weight. As we already pointed out, it is a good source of GAGs, especially chondroitin sulfate. Other studies were focused on extracting collagen from cartilage in various sturgeon species. These research studies focused on optimizing the parameters of the extraction process, on characterizing the extracted collagen by evaluating the fibril-forming characteristics and on evaluating collagen-derived functional peptides [97,98,99,100,101,102].

The method used to extract type II collagen from sturgeon cartilage was optimized. The ideal conditions (pyrolysis temperature of 120 °C for 30 min, an enzyme concentration of 8000 U/g protein, a hydrolysis duration of 4 h, a pH of 8 and a hydrolysis temperature of 50 °C) led to 10.85% degree of hydrolysis for type II collagen, with a protein content of 70.7% [97]. 

Type II collagen was extracted from cartilage of a hybrid (*A. schrencki* × *H. dauricus*). This collagen retains the natural and complete triple-helix structure and has the potential to replace collagen from mammals [98]. Meng et al. (2023) extracted both chondroitin sulfate (CS) and type II collagen (Col II) from Russian sturgeon (*A. gueldenstaedtii*) notochord. The study proposed a new extraction method in order to obtain CS and undenatured Col II simultaneously with significant yields (5.34 ± 0.74% and 45.25 ± 5.25%, respectively). The Col II retains the triple-helical structure and the CS accelerated the completion of Col II self-assembly. The study suggested not only that the sturgeon notochord is a valuable source of CS and Col II but also that the new extraction method improves the utilization rate of sturgeon notochord. Moreover, CS and Col II derived from sturgeon notochord, based on the displayed characteristics, have the potential for use in biomedical materials [99]. The structural properties and biological activities of collagens extracted from four by-products (skin, fin, cartilage and notochord) of Russian sturgeon were evaluated. Cartilage and notochord collagens were characterized as type II collagen that could only be self-assembled into fibrils at a low phosphate ion concentration [100].

After extracting Col II from sturgeon cartilage characterized by a triple-helix structure with a repeating tripeptide unit Gly–X–Y, where X and Y are generally proline or hydroxyproline, the effects of administrating this compound on rheumatoid arthritis (RA) were evaluated in rats. The results were promising, showing the reduction of gene expression for the TNF-α, IL-1β, COX-2, MCP-1 and TLR-4 genes and a decrease in the protein expression of TNF-α, IL-1β and RF. Therefore, CII extracted from sturgeon cartilage demonstrated improving effects on RA [101].

After collagen extraction from bone and cartilage, this is enzymatically hydrolyzed to release physiologically active peptides. For example, for the collagen extracted from sturgeon bone, the enzymatic hydrolysis (with enzyme concentration of 1.5 × 105 IU/g, the ratio of bone powder to water 1:25, the enzymatic hydrolysis time of 5 h, the enzymatic hydrolysis temperature of 50 °C and the ratio of alkaline protease: neutral protease enzyme activity of 1:2) led to a yield of bone collagen peptide of about 72% [10]. 

The collagen peptides that were recovered after the hydrolysis with papain of the collagen extracted from different by-products of Russian sturgeon showed antioxidant activity. The notochord and skin collagen peptide had better capacity for scavenging reactive oxygen species (ROS) than the ones isolated from fin and cartilage [100]. 

A similar study was conducted in Siberian sturgeon (*A. baerii*). The cartilages were degreased, mineralized and separately hydrolyzed by five types of proteases. The collagen hydrolysate (SCH) generated by alcalase treatment showed the antioxidant capacity. Thirteen antioxidant peptides were isolated from SCH and three of these showed the highest antioxidant activity, inhibiting the lipid peroxidation and showing protective functions on H_2_O_2_-damaged plasmid DNA. Furthermore, these functional peptides derived from collagen displayed significant cytoprotection of the cells from human umbilical vein endothelial cells (HUVECs) against H_2_O_2_ injury. The protective capacity was demonstrated by regulating the endogenous antioxidant enzymes superoxide dismutase (SOD) and glutathione peroxidase (GSH-Px) to decrease the content of ROS. Such types of functional peptides derived from collagen isolated from different sturgeon by-products may act as antioxidant additives for generating health products to treat chronic diseases caused by oxidative stress [102].

The studies conducted to date regarding collagen and collagen peptides provide basic data for the application of this biomolecules in the food and biomedical material industries in order to increase the value of sturgeon by-products.

#### 4.2.2. Skin/Scales

The quantity of sturgeon skin accounts for 5–7% of the total fresh fish weight, but unfortunately this product is largely discarded in sturgeons. Studies were conducted on utilizing skin as a source of collagen, focusing on optimizing the conditions of extraction and on evaluating the properties of collagen and collagen-derived peptides. 

Collagen extracted from skin was utilized as the main source for isolating dpp-IV inhibitory peptide. In order to prepare collagen-derived peptides from skin with a maximum yield and optimum qualities, different methods of hydrolysis (alkaline protease hydrolysis, acid hydrolysis, enzymatic extraction, etc.) were tested. For example, acid and enzymatic extraction led to different yields of acid soluble collagen (ASC) (9.98%) and pepsin soluble collagen (PSC) (9.08%) [103,104].

The skin PSC from Russian sturgeon was characterized as type I collagen that could be self-assembled into fibrils in a wide range of phosphate ion concentrations. The fibril-forming ability of sturgeon type I collagen was higher than that of porcine type I collagen. The fibril diameter of type I collagen was higher than that of type II collagen. The antioxidant activity of skin collagen peptides was higher than that of the fin and cartilage collagen peptides [100]. 

Skin is also a good source of gelatin. The properties and functionality of fish gelatin are influenced by the fish species, the specific organs used, pretreatment methods, and extraction conditions. Gelatin is typically extracted from fish tissues that have been treated with either alkali or acid under a hot-water extraction process. Gelatin extracted following an acid pretreatment is referred to as type A, while gelatin resulting from an alkali pretreatment is known as type B. Both acid and alkali pretreatments are essential to eliminate unwanted odors and colors, swell the tissues by weakening the collagen molecules’ triple helix, and reduce the extraction time [105].

Nikoo et al. (2014) isolated gelatin from Amur sturgeon (*A. schrenckii*) skin, recovering this molecule in a range of 9.42–12.47% (wet weight), and then tested the cryoprotective effects of a tetrapeptide obtained from gelatin hydrolysis [106]. Another method for gelatin isolation from the head of Kalamtra sturgeon (*H. dauricus* × *A. scherenkii* × *A. transmontanus*) was proposed by Islam et al. (2020) by optimizing the conditions for pretreatment and extraction that led to yields of type A and B gelatins of 5.01 and 7.25% (dry gelatin weight/wet sample weight), respectively. Thus, the process of extraction can be restructured to an industrial scale, making the sturgeon by-products valuable sources of gelatin with application in the foods, cosmetics and biomedical industries [107].

#### 4.2.3. Viscera

As a by-product of sturgeon processing, sturgeon viscera can cause environmental pollution if discarded improperly. To address this, some researchers have explored ways to enhance the comprehensive utilization value of sturgeon viscera. This by-product is a good source of proteins, so different variants of extraction methods of protein hydrolysates from Siberian sturgeon viscera were tested in order to obtain the maximum yield of a good quality reflected by the composition of EAA and NEAA. Thus, among five different enzymes, alcalase provided the best results, with the highest protein recovery rate of 83.64% [86].

Similar to other sturgeon by-products, viscera are a good source of collagen. PSC was extracted from the sturgeon swim bladder from Amur sturgeon (*A. schrenckii*). When compared with PSC isolated from the carp (*Ctenopharyngodon idella*) swim bladder, the collagen extracted from the sturgeon bladder was available in a significantly higher amount and had better thermal stability [108]. For the same sturgeon species, bladder functional peptides were isolated, identified and tested for their antioxidant activity and physicochemical properties. The results indicated that the optimal enzymatic conditions were as follows: alkaline protease with a solid-to-liquid ratio of 1:20, an incubation time of 4 h, a temperature of 55 °C and an enzyme dosage of 5000 U/g. Three different molecular weight fractions (F1, F2, and F3) were recovered by ultrafiltration, with F3 demonstrating a superior antioxidant activity. Peptide sequence analysis revealed that F3 contained antioxidant peptides (MFGF, GPPGPRGPPGL, and GPGPSGERGPPGPM) and exhibited inhibitory activities on the angiotensin-converting enzyme and dipeptidyl peptidase III/IV (FRF, FPFL, and LPGLF) [109]. 

#### 4.2.4. Sturgeon Oil

Sturgeon oil is rich in highly unsaturated fatty acids, surpassing those found in most terrestrial animals and plants. Moreover, compared to other freshwater fish, sturgeon oil has a much higher content of PUFA. Hao et al. (2014) assessed the effects of different extraction methods on the composition and storage stability of sturgeon oil. Five methods (supercritical fluid extraction –SFE, amino, wet reduction and enzymatic extraction) were compared, revealing that the recovery of sturgeon oil with SFE is high, reaching up to 97.25%, followed by enzymatic extraction. SFE led to a higher oil yield, better color attributes and higher oxidative stability compared to other traditional extraction methods, such as enzymatic extraction, amino and wet reduction. The wet reduction and enzymatic methods led to a high levels of organic acids and the amino method resulted in amine compounds in sturgeon oil. After storage at 4 °C for 33 days, the aldehyde content in oil extracted by the enzymatic extraction and wet reduction methods was twice as high as that obtained by the other methods. In conclusion, SFE possesses some advantages over other extraction methods and is recommended for sturgeon oil extraction [110].

Another study focused on optimizing the enzymatic extraction process of sturgeon fish oil and exploring its interventional effect on non-alcoholic fatty liver disease (NAFLD) by using a murine model. Under the optimal extraction conditions (1500 U/g papain, the ratio of solid to liquid 1:1 (mL/g), pH 7, and the enzymatic hydrolysis time at 45 °C for 2 h), the extraction rate of sturgeon oil was 82.94. The saturated fatty acids and unsaturated fatty acids content of extracted sturgeon oil were 23.56% and 74.71%, respectively, with a high content of n-3 unsaturated fatty acids. Subsequently, following administration of the oil to the mice, some serum parameters (total cholesterol, triglycerides, low-density lipoprotein, alanine transferase and aspartate transferase) significantly increased while others (high-density lipoprotein) significantly decreased in the high-fat group. Thus, the sturgeon oil can significantly improve dyslipidemia and other manifestations of NAFLD and can be used as an alternative product of deep-sea fish oil [111]. Sturgeon oil has also proven efficient in atopic dermatitis (AD) treatment and improving the intestinal imbalance caused by a high-fat diet [112,113].

## 5. Conclusions

Sturgeon caviar, meat and by-products are rich in essential nutrients, including high-quality proteins, omega-3 fatty acids, vitamins and minerals. These components exhibit, among others, anti-inflammatory and antioxidant properties and convey various health benefits, such as improved cardiovascular health and cognitive function. The sturgeon caviar industry significantly contributes to the global economy, mostly in regions where sturgeon farming and caviar production are prominent, supporting local economies and creating job opportunities in aquaculture, processing and related sectors.

Beyond caviar and meat, sturgeon by-products, such as cartilage, skin, viscera, etc., have potential applications in the pharmaceutical, nutraceutical, food, cosmetic and medical sectors. This diversification increases the overall value of sturgeon aquaculture and promotes a zero-waste approach.

Even though, in the last decade, tremendous progress has been unregistered, constant research and innovation in sustainable sturgeon farming practices are crucial. In sturgeon aquaculture, a direction is orientated to making caviar more accessible to a larger consumer base without compromising quality. In this regard, research was focused on optimizing the feeding diet and environmental conditions. Sustainable caviar production, together with reinforcement of international and local regulatory frameworks to combat illegal poaching and trade, has an important impact on wild sturgeon protection and establishing ethical practices within the industry. Moreover, exploring new applications for sturgeon by-products can open up supplementary profits streams and reduce waste. Joint efforts between the aquaculture industry and other sectors, such as pharmaceuticals and cosmetics, can drive innovation and market growth. A limitation of these studies is caused by the deficient standardization of the methods for extracting and processing sturgeon by-products, which can result in the varying quality and efficacy of the derived products. Inconsistent use of analytical techniques to assess chemical and biochemical properties hinders the comparability and reproducibility of research findings. Also, the cost of extracting and processing sturgeon by-products can be high, and there is limited research on making these processes economically viable on a large scale. The absence of advanced and cost-effective processing technologies limits the ability to fully exploit the chemical and biochemical potential of sturgeon by-products. Therefore, the research might be orientated toward comprehensive analysis of the functional properties of sturgeon by-products, such as the bioactivity, shelf-life stability and performance in specific applications. 

In conclusion, while sturgeon caviar, meat, and by-products hold significant importance in terms of the nutritional and economic value, sustainable practices and innovative approaches are essential to ensure the industry’s future growth and ecological balance. Despite the fact that there is significant potential in terms of the utilization of sturgeon by-products, research in this area is constrained by several factors. Addressing these limitations requires coordinated efforts, interdisciplinary collaboration and advancements in processing technologies and regulatory frameworks. 

## Figures and Tables

**Table 1:** Proximate composition of raw eggs/caviar and egg size in different sturgeon species.

Scientific Name/Common Name	Origin	Type of Sample *	Water (g/100 g Wet Weight)	Protein (g/100 g Wet Weight)	Lipids (g/100 g Wet Weight)	Carbohydrates (g/100 g Wet Weight)	d (mm)	References
*A. baerii*/Siberian sturgeon	Farmed (France)	Caviar	53.02 ± 0.23	26.21 ± 1.14	10.90 ± 0.07	-	3.06 ± 0.25	[26]
	Farmed (Italy)	Eggs	59.50 ± 0.90	23.80 ± 0.80	14.90 ± 0.90	-	-	[31]
	Farmed (Italy)	Caviar	57.30 ± 2.50	23.90 ± 2.20	14.90 ± 0.70	-	-	[31]
	Farmed (China)	Caviar	51.80 ± 0.98	23.98 ± 0.78	14.23 ± 0.71	-	-	[32]
	Farmed (Poland)	Eggs	64.00	19.82 ± 1.11	10.46 ± 1.09		2.51 ± 0.13	[33]
*A. gueldenstaedtii*/Russian sturgeon	Wild (Danube River/Romania)	Caviar	41.90 ± 3.21	29.32 ± 0.92	17.13 ± 0.76	-	3.24 ± 0.15	[26]
	Wild (Caspian Sea)	Caviar	50.90 ± 2.32	27.01 ± 1.12	14.05 ± 1.20	-	3.01 ± 0.24	[26]
	Unspecified (Iran)	Caviar	52.00 ± 0.80	24.00 ± 2.90	14.60 ± 2.40	4.60 ± 1.94	-	[34]
	Farmed (Italy)	Eggs	53.90 ± 2.00	24.10 ± 1.00	19.70 ± 1.80	-	-	[31]
	Farmed (Italy)	Caviar	52.70 ± 2.20	24.70 ± 1.20	19.10 ± 3.20	-	-	[31]
*A. persicus*/Persian sturgeons	Unspecified (Iran)	Caviar	51.50 ± 0.50	24.20 ± 1.30	14.70 ± 0.50	5.40 ± 0.60	-	[34]
*A. ruthenus*/sterlet sturgeon	Farmed (Korea)	Caviar	51.32	25.43	13.21	-	-	[35]
*A. schrenckii*/Amur sturgeon	Farmed (China)	Caviar	48.65 ± 0.90	24.27 ± 1.93	15.99 ± 0.93	-	-	[32]
*A. stellatus*/stellate sturgeon	Wild (Danube River/Romania)	Caviar	38.52 ± 3.01	31.13 ± 1.53	16.96 ± 0.83	-	2.85 ± 0.34	[26]
	Wild (Caspian Sea)	Caviar	51.81 ± 3.59	27.59 ± 1.72	14.58 ± 1.20	-	2.67 ± 0.19	[26]
*A. transmontanus* (white sturgeon)	Farmed (Italy)	Eggs	57.10 ± 1.30	24.90 ± 0.60	16.10 ± 1.20	-	-	[31]
	Farmed (Italy)	Caviar	54.60 ± 0.90	24.00 ± 1.30	17.70 ± 1.20	-	-	[31]
*H. huso* (beluga sturgeon)	Wild (Danube River/Romania)	Caviar	37.10	30.49	19.41	-	3.47 ± 0.23	[26]
	Wild (Caspian Sea)	Caviar	52.93 ± 1.23	28.44 ± 1.12	15.16 ± 1.09	-	3.75 ± 0.27	[26]
	Wild (Caspian Sea/Iran)	Eggs	64.10 ± 2.10	15.11 ± 0.97	14.87 ± 1.00	-		[36]
	Farmed (Iran)	Eggs	64.83 ± 0.46	14.56 ± 0.20	14.55 ± 0.60	-		[36]
	Wild (Caspian Sea/Iran)	Eggs	56.21 ± 4.26	25.43 ± 2.83	14.80 ± 1.74	-	-	[37]
	Farmed (Iran)	Eggs	57.29 ± 3.23	23.81 ± 3.88	15.67 ± 2.16	-	3.00–5.00	[37]
	Wild (Caspian Sea)	Caviar	51.26 ± 0.21	26.56 ± 0.11	16.06 ± 0.16	-		[38]
	Farmed (Iran)	Caviar	51.40 ± 0.28	26.37 ± 0.65	16.35 ± 0.18	-		[38]
	Unspecified (Iran)	Caviar	48.40 ± 2.20	24.70 ± 0.30	15.90 ± 2.00	6.90 ± 1.40	-	[34]
Hybrid *(A. schrenckii × H. dauricus)*	Farmed (China)	Caviar	47.72 ± 1.62	25.55 ± 1.82	16.22 ± 1.12	-	-	[32]

* Caviar is used for salted and processed roe, while eggs represent the raw roe of sturgeons. d—medium diameter.

**Table 2:** Proximate composition of meat from different sturgeon species.

Scientific Name/Common Name	Origin	Age	Water (g/100 g Wet Weight)	Protein (g/100 g Wet Weight)	Lipids (g/100 g Wet Weight)	References
*A. baerii*/Siberian sturgeon	Farmed (Italy)	Adult	75.50 ± 1.60	17.60 ± 0.50	5.60 ± 1.70	[31]
	Farmed (China)	Juveniles	74.00 ± 1.10–77.40 ± 0.70	15.70 ± 0.30–16.50 ± 0.10	5.80 ± 0.50–8.60 ± 1.20	[58]
	Farmed (Italy)	3.5–4.5 Y	72.11 ± 0.58	19.47 ± 0.24	7.76 ± 0.59	[59]
	Farmed	1 Y+	75.20 ± 0.75	14.30 ± 0.72	9.50 ± 0.39	[60]
*A. gueldenstaedtii*/Russian sturgeon	Farmed (Turkey)	Juveniles	76.21 ± 0.09–78.24 ± 0.05	10.71 ± 0.04–11.63 ± 0.02	4.65 ± 0.03–6.05 ± 0.07	[61]
	Farmed (China)	Juveniles	78.10 ± 0.85–79.74 ± 0.38	16.12 ± 0.42–16.96 ± 0.21	2.54 ± 0.21–2.60 ± 0.03	[62]
	Farmed (Turkey)	Juveniles	74.72 ± 0.10–78.18 ± 0.23	18.44 ± 1.53–20.78 ± 0.24	3.41 ± 0.21–6.64 ± 0.42	[63]
*A. naccarii*/Adriatic sturgeon	Farmed (Italy)	3.5–4.5 Y	69.81 ± 0.60	18.64 ± 0.27	10.64 ± 0.76	[59]
*A. persicus*/Persian sturgeon	South coast of the Caspian Sea (Iran)	Adult	63.20 ± 0.41	21.40 ± 0.38	13.10 ± 1.13	[64]
*A. transmontanus*/white sturgeon	Farmed (Italy)	Adult	75.20 ± 3.30 * 77.70 ± 1.10 **	19.60 ± 0.80 * 18.60 ± 0.50 **	3.90 ± 2.50 * 2.60 ± 0.80 **	[31]
	Farmed (Italy)	3.5–4.5 Y	75.55 ± 0.54	19.57 ± 0.28	4.49 ± 0.41	[59]
	Farmed (Italy)	Adult (3–5 Y)	76.35 ± 0.98–77.41 ± 1.05	18.66 ± 0.59–18.98 ± 0.73	2.50 ± 1.04–3.36 ± 0.74	[65]
*H. huso*/beluga sturgeon	Farmed (Iran)	Adult	70.75 ± 1.34	18.45 ± 0.49	4.25 ± 0.91	[27]
Hybrid (*A. baerii* × *A. medirostris*)	Farmed (Poland)	1 Y+	77.40 ± 0.44	15.20 ± 0.41	6.40 ± 0.47	[60]
Hybrid *A. baerii* × (*A. baerii* × *A. medirostris*)	Farmed Poland	1 Y+	73.52 ± 0.55	16.51 ± 0.38	8.90 ± 0.47	[60]

The values reported as an interval represent the lowest and highest obtained for different diet formulations; the ones reported as a single value were obtained for uniform feeding regimens and environmental conditions; * female (for caviar production), ** male (for meat production).

**Table 3:** Comparison of major classes of FA (g/100 g of wet weight) in different farmed sturgeon species.

Fatty Acids	*H. huso*		*Acipenser* spp.	*A. baerii*			*A. naccarii*	*A. transmontanus*			*A. gueldenstaedtii*	*A. naccarii × A. baerii*	*A. baerii ×* *A. medirostris*	*A. baerii* × (*A. baerii* × *A. medirostris*)
ΣSFA	29.93	33.61	25.99	24.75	27.00	19.20	26.34	26.88	31.90 ^a^ 29.95 ^b^	23.40 ^e^ 24.50 ^f^	23.39 ^c^ 24.58 ^d^	26.76	25.84	26.71
ΣMUFA	41.93	32.46	46.00	44.89	39.90	45.50	47.7	45.39	69.30 ^a^* 70.10 ^b^*	42.70 ^e^ 31.30 ^f^	38.4 ^c^ 27.86 ^d^	37.92	46.15	46.21
ΣPUFA	32.44	21.30	22.4	25.21	32.90	35.30	20.17	21.81	69.30 ^a^* 70.10 ^b^*	33.90 ^e^ 44.20 ^f^	37.62 ^c^ 47.56 ^d^	34.7	28.01	27.08
Σn-3	7.24	17.48	18.09	19.98	20.20	15.00	16.66	17.62	21.10 ^a^ 23.40 ^b^	18.50 ^e^ 24.70 ^f^	14.13 ^c^ 14.36 ^d^	30.19	22.68	19.58
Σn-6	25.31	3.54	4.31	5.23	9.99	20.40	3.51	4.18	8.55 ^a^ 9.10 ^b^	15.40 ^e^ 19.50 ^f^	23.49 ^c^ 33.21 ^d^	4.48	5.32	6.59
n-3/n-6	0.28	4.94	4.26	3.83	2.04	0.70	4.74	4.21	2.47 ^a^ 2.64 ^b^	1.20 ^e^ 1.40 ^f^	0.6 ^c^ 0.43 ^d^	6.74	4.26	2.97
C20:5n-3 (EPA)	0.55	4.65	5.63	6.54	-	3.90	4.81	5.55	-	5.70 ^e^ 8.60 ^f^	1.99 ^c^** 2.31 ^d^**	9.40	6.04	5.46
C22:6n-3	2.17	12.41	9.18	9.70	-	7.30	8.77	9.06	-	9.70 ^e^ 12.30 ^f^	-	15.01	11.28	9.34
Reference	[70]	[66]	[69]	[59]	[71]	[31]	[59]	[59]	[65]	[31]	[63]	[72]	[60]	[60]

^a^ group 5 kg, ^b^ group 10 kg, ^c^ diet 1 (enriched with canola oil), ^d^ diet 2 (enriched with sun flower oil), ^e^ female (for caviar production), ^f^ male (for meat production), * total content of unsaturated fatty acids (ΣMUFA + PUFA), ** DHA/EPA.

**Table 4:** Overview of sturgeon by-products: valuable components and their uses.

By-Product	Valuable Components	Possible Uses
Heads	Proteins, peptides, lipids, collagen, gelatin, minerals (calcium)	Food industry, fish meal, food grade hydrolysates, pet food, nutraceuticals, cosmetics
Frames (bones, flesh, fins)	Proteins, peptides, lipids, collagen, gelatin, minerals (calcium)	Food industry, fish meal, food grade hydrolysates, pet food, nutraceuticals, cosmetics
Cartilage	Proteins, peptides, collagen, gelatin,	Food industry, health supplements, cosmetics, organic fertilizer, animal feed additive
Bladder	Isinglass	Foods and drinks industry (for the clarification of beer and wine)
Skin	Collagen, gelatin, protein, peptides, lipids, minerals	Fashion industry (leather), food industry, pharmaceuticals, cosmetics, fish meal, fish oil
Viscera	Proteins, peptides, lipids, enzymes	Fish oil, fish meal, organic fertilizer, animal feed additive, biofuel

## Data Availability

No new data were created.

## References

[1] Bemis W.E., Kynard B. (1997). Sturgeon rivers: An introduction to Acipenseriform biogeography and life history. Environ. Biol. Fish..

[2] Bemis W.E., Findeis E.K., Grande L. (1997). An overview of Acipenseriformes. Environ. Biol. Fish..

[3] Fish Base. https://www.fishbase.se/photos/UploadedBy.php?autoctr=42814&win=uploaded.

[4] Fish Base. https://www.fishbase.se/photos/UploadedBy.php?autoctr=42838&win=uploaded.

[5] Billard R., Lecointre G. (2001). Biology and conservation of sturgeon and paddlefish. Rev. Fish Biol. Fish..

[6] Nelson T.C., Doukakis P., Lindley S.T., Schreier A.D., Hightower J.E., Hildebrand L.R., Whitlock R.E., Webb M.A.H. (2013). Research Tools to Investigate Movements, Migrations, and Life History of Sturgeons (*Acipenseridae*), with an Emphasis on Marine-Oriented Populations. PLoS ONE.

[7] IUCN Red List of Threatened Species. https://www.iucn.org/content/sturgeon-more-critically-endangered-any-other-group-species.

[8] Bronzi P., Chebanov M., Michaels J.T., Wei Q., Rosenthal H., Gessner J. (2019). Sturgeon meat and caviar production: Global update. J. Appl. Ichthyol..

[9] Vasilyeva L.M., Elhetawy A.I.G., Sudakova N.V., Astafyeva S.S. (2019). History, current status and prospects of sturgeon aquaculture in Russia. Aquac. Res..

[10] Chen R., Liu Z., Wang J., Jin W., Abdu H.I., Pei J., Wang Q., Abd El-Aty A.M. (2022). A review of the nutritional value and biological activities of sturgeon processed byproducts. Front. Nutr..

[11] Google Scholar. https://scholar.google.com/scholar?q=%22sturgeon+aquaculture%22&hl=en&as_sdt=0%2C5&as_ylo=2000&as_yhi=2024.

[12] Openknowledge.fao.org/FAO Report Sustainable Fisheries and Aquaculture for Food Security and Nutrition. A Report by the High Level Panel of Experts on Food Security and Nutrition. http://www.fao.org/3/a-i3844e.pdf.

[13] White P., Hasan M.R., New M.B. (2013). Environmental consequences of poor feed quality and feed management. On-Farm Feeding and Feed Management in Aquaculture.

[14] Anderson W.G., Schreier A., Crossman J.A. (2022). Conservation aquaculture—A stocking story. Fish Physiol..

[15] Stevens J.R., Newton R.W., Tlusty M., Little D.C. (2018). The rise of aquaculture by-products: Increasing food production, value, and sustainability through strategic utilisation. Mar. Policy.

[16] Laktuka K., Kalnbalkite L., Logins K., Lauka D. (2023). Towards the Sustainable Intensification of Aquaculture: Exploring Possible Ways Forward. Sustainability.

[17] Bledsoe G.E., Bledsoe C.D., Rasco B. (2003). Caviars and fish roe products. Crit. Rev. Food Sci. Nutr..

[18] CITES Trade Database. https://trade.cites.org/en/cites_trade/download/view_results?filters%5Btime_range_start%5D=2022&filters%5Btime_range_end%5D=2024&filters%5Bexporters_ids%5D%5B%5D=all_exp&filters%5Bimporters_ids%5D%5B%5D=all_imp&filters%5Bsources_ids%5D%5B%5D=106&filters%5Bpurposes_ids%5D%5B%5D=123&filters%5Bterms_ids%5D%5B%5D=12&filters%5Btaxon_concepts_ids%5D%5B%5D=&filters%5Breset%5D=&filters%5Bselection_taxon%5D=taxonomic_cascade&web_disabled=&filters[report_type]=comptab.

[19] Ghelichi S., Hajfathalian M., Bekhit A.E.D.A. (2022). Caviar: Processing, composition, safety, and sensory attributes. Fish Roe.

[20] https://globalseafoods.com/blogs/news/osetra-caviar-vs-beluga-caviar-which-one-is-better?_pos=1&_sid=effce6b44&_ss=r.

[21] Chebanov M.S., Billard R. (2001). The culture of sturgeons in Russia: Production of juveniles for stocking and meat for human consumption. Aquat. Living Resour..

[22] Conte F.S., Doroshov S.I., Lutes P.B., Strange E.M. (1988). Hatchery Manual for the White Sturgeon (Acipenser transmontanus Richardson), With Application to Other North American Acipenseridae.

[23] Wei Q., Ke F., Zhang J., Zhuang P., Luo J., Zhou R., Yang W., Birstein V.J., Waldman J.R., Bemis W.E. (1997). Biology, fisheries, and conservation of sturgeons and paddlefish in China. Sturgeon Biodiversity and Conservation.

[24] Gessner J., Wirth M., Kirschbaum F., Krüger A., Patriche N., Fredrich F. (2002). Caviar composition in wild and cultured sturgeons--impact of food sources on fatty acid composition and contaminant load. J. Appl. Ichthyol..

[25] Omoto N., Arai K., Adachi S. (2005). Growth and reproductive performance of hybrid sturgeons (*Huso dauricus × Acipenser schrenckii*) in captivity. Aquaculture.

[26] Wirth M., Kirschbaum F., Gessner J., Kruger A., Patriche N., Billard R. (2000). Chemical and biochemical composition of caviar from different sturgeon species and origins. Nahr.-Food.

[27] Pourshamsian K., Ghomi M.R., Nikoo M. (2012). Fatty acid and proximate composition of farmed great sturgeon (*Huso huso*) affected by thawing methods, frying oils and chill storage. Adv. Stud. Biol..

[28] Khan S.U., Lone A.N., Khan M.S., Virani S.S., Blumenthal R.S., Nasir K., Miller M., Michos E.D., Ballantyne C.M., Boden W.E. (2021). Effect of omega-3 fatty acids on cardiovascular outcomes: A systematic review and meta-analysis. eClinicalMedicine.

[29] Wei B.Z., Li L., Dong C.W., Tan C.C., Xu W. (2023). The Relationship of Omega-3 Fatty Acids with Dementia and Cognitive Decline: Evidence from Prospective Cohort Studies of Supplementation, Dietary Intake, and Blood Markers. Am. J. Clin. Nutr..

[30] Schmidt C.V., Raza H., Olsen K., Mouritsen O.G. (2024). Proximate nutritional composition of roe from fish, crustaceans, mussels, echinoderms, and cephalopods. Int. J. Gastron. Food Sci..

[31] Lopez A., Vasconi M., Bellagamba F., Mentasti T., Moretti V.M. (2020). Sturgeon meat and caviar quality from different cultured species. Fishes.

[32] Gong Y., Huang Y., Gao L., Lu J., Hu Y., Xia L., Huang H. (2013). Nutritional composition of caviar from three commercially farmed sturgeon species in China. J. Food Nutr. Res..

[33] Kowalska-Goralska M., Formicki K., Dobrzanski Z., Wondołowska-Grabowska A., Skrzynska E., Korzelecka-Orkisz A., Nędzarek A., Tanski A. (2020). Nutritional composition of Salmonidae and Acipenseridae fish eggs. Ann. Anim. Stud..

[34] Mol S., Turan S. (2008). Comparison of proximate, fatty acid and amino acid compositions of various types of fish roes. Int. J. Food Prop..

[35] Park K.S., Kang K.H., Bae E.Y., Baek K.A., Shin M.H., Kim D.U., Kang H.K., Kim K.J., Choi Y.J., Im J.S. (2015). General and biochemical composition of caviar from sturgeon (*Acipenser ruthenus*) farmed in Korea. Int. Food Res. J..

[36] Ovissipour M., Rasco B. (2011). Fatty acid and amino acid profiles of domestic and wild beluga (*Huso huso*) roe and impact on fertilization ratio. J. Aquacult. Res. Dev..

[37] Hamzeh A., Moslemi M., Karaminasab M., Khanlar M.A., Faizbakhsh R., Navai M.B., Tahergorabi R. (2015). Amino acid composition of roe from wild and farmed Beluga sturgeon (*Huso huso*). J. Agric. Sci. Technol..

[38] Barimani S., Hedayatifard M., Motamedzadegan A., Bozorgnia A. (2021). Changes of amino acids and proximate compositions in freshwater farmed beluga sturgeon (*Huso huso*) caviar. Iran. J. Fish. Sci..

[39] Farag M., Abib B., Tawfik S., Shafik N., Khattab A. (2021). Caviar and fish roe substitutes: Current status of their nutritive value, bio-chemical diversity, authenticity and quality control methods with future perspectives. Trends Food Sci. Technol..

[40] Caprino F., Moretti V.M., Bellagamba F., Turchini G.M., Busetto M.L., Giani I., Paleari M.A., Pazzaglia M. (2008). Fatty acid composition and volatile compounds of caviar from farmed white sturgeon (*Acipenser transmontanus*). Anal. Chim. Acta.

[41] DePeters E.J., Puschner B., Taylor S.J., Rodzen J.A. (2013). Can fatty acid and mineral compositions of sturgeon eggs distinguish between farm-raised versus wild white (*Acipenser transmontanus*) sturgeon origins in California? Preliminary report. Forensic Sci. Int..

[42] Czesny S., Dabrowski K., Christensen J.E., Van Eenennaam J., Doroshov S. (2000). Discrimination of wild and domestic origin of sturgeon ova based on lipids and fatty acid analysis. Aquaculture.

[43] Shin J.H., Oliveira A.C.M., Rasco B.A. (2010). Quality attributes and microbial storage stability of caviar from cultivated white sturgeon (*Acipenser transmontanus*). J. Food Sci..

[44] Gessner J., Würtz S., Kirschbaum F., Wirth M. (2008). Biochemical composition of caviar as a tool to discriminate between aquaculture and wild origin. J. Appl. Ichthyol..

[45] Wirth M., Kirschbaum F., Gessner J., Williot P., Patriche N., Billard R. (2002). Fatty Acid Composition in Sturgeon Caviar from Different Species: Comparing Wild and Farmed Origins. Int. Rev. Hydrobiol..

[46] Cardinal M., Williot P., Nonnotte G., Chebanov M. (2018). Caviars: How to Describe and Compare Their Qualities? The Sensorial Approach. Chebanov: The Siberian Sturgeon (Acipenser baerii, Brandt, 1869) Volume 2—Farming.

[47] Lukiw W.J., Bazan N.G. (2008). Docosahexaenoic Acid and the Aging Brain. J. Nutr..

[48] Yamanaka T., Namura M., Koseki K., Bito T., Umebayashi Y., Watanabe F. (2022). Characterization of vitamin B12 compounds from commercially available fish roe products. Fish. Sci..

[49] Chebanov M., Rosenthal H., Gessner J., Van Anrooy R., Doukakis P., Pourkazemi M., Williot P. (2011). Sturgeon Hatchery Practices and Management for Release-Guidelines FAO Fisheries and Aquaculture Technical Paper No 570. https://www.fao.org/4/i2428e/i2428e.pdf.

[50] Raposo A., Alturki H.A., Alkutbe R., Raheem D. (2023). Eating Sturgeon: An Endangered Delicacy. Sustainability.

[51] Monavar M., Alimardani R., Omid M., Wold J.P. (2012). Prediction of species and freshness of Caspian caviar during storage by front-face fluorescence spectroscopy. Int. J. Agric. Technol..

[52] EUMOFA (2018). The Caviar Market. https://eumofa.eu/documents/20124/55247/2021+-+The+Caviar+Market.pdf.

[53] EUMOFA (2023). Sturgeon Meat and Other by-Products of Caviar. https://eumofa.eu/documents/20124/35725/Sturgeon+meat.pdf/5e78102f-670e-bae9-521a-a2d764e59aa3?t=1675868036405.

[54] Fletcher N. (2010). Caviar: A Global History.

[55] Global Seafood Alliance. https://www.globalseafood.org/advocate/as-sturgeon-farming-grows-demand-concerns-emerge/.

[56] EUMOFA The EU Fish Market. https://eumofa.eu/documents/20124/35668/EFM2023_EN.pdf/95612366-79d2-a4d1-218b-8089c8e7508c?t=1699541180521.

[57] Chebanov M., Williot P., Williot P., Nonnotte G., Chebanov M. (2018). An Assessment of the Characteristics of World Production of Siberian Sturgeon Destined to Human Consumption. Chebanov: The Siberian Sturgeon (Acipenser baerii, Brandt, 1869) Volume 2—Farming.

[58] Xue M., Yun B., Wang J., Sheng H., Zheng Y., Wu X., Qin Y., Li P. (2012). Performance, body compositions, input and output of nitrogen and phosphorus in Siberian sturgeon, Acipenser baerii Brandt, as affected by dietary animal protein blend replacing fishmeal and protein levels. Aquac. Nutr..

[59] Badiani A., Stipa S., Nanni N., Gatta P.P., Manfredini M. (1997). Physical indices, processing yields, compositional parameters and fatty acid profile of three species of cultured sturgeon (genus Acipenser). J. Sci. Food Agric..

[60] Jankowska B., Kolman R., Szczepkowski M., Żmijewski T. (2005). Production value, chemical composition and colour of fillets of the reciprocal hybrid of Siberian sturgeon with green sturgeon (*Acipenser baeri* Br × (*Acipenser baeri* × *Acipenser medirostris* Ayres). Czech J. Anim. Sci..

[61] Şener E., Yildiz M., Savaş E. (2005). Effects of Dietary Lipids on Growth and Fatty Acid Composition in Russian Sturgeon (*Acipenser gueldenstaedtii*) Juveniles. Turk. J. Vet. Anim. Sci..

[62] Huang H.L., Lu J.X., Gao L.J., Huang Y.Q., Gong Y.Y. (2016). Substitution of krill meal for fish meal in feed for Russian Sturgeon, *Acipenser gueldenstaedtii*. Isr. J. Aquac. -Bamidgeh.

[63] Tiril S.U., Dernekbasi S., Karayucel I., Kerim M., Akyuz A.P. (2016). Effects of Canola and Safflower Oil Supplementation in Diets, on Growth Performance and Fatty Acid Composition of Russian Sturgeon (*Acipenser gueldenstaedtii* Brandt, 1833). Isr. J. Aquac. Bamidgeh.

[64] Babaei S., Abedian-Kenari A., Hedayati M., Yazdani-Sadati M.A. (2017). Growth response, body composition, plasma metabolites, digestive and antioxidant enzymes activities of Siberian sturgeon (*Acipenser baerii*, Brandt, 1869) fed different dietary protein and carbohydrate: Lipid ratio. Aqua Res..

[65] Paleari M.A., Beretta G., Grimaldi P., Vaini F. (1997). Composition of muscle tissue of farmed white sturgeon (*Acipenser transmontanus*) with particular reference to lipidic content. J. Appl. Ichthyol..

[66] Yalçin K., Hülya T., Erdem E.M. (2008). Fatty acid and amino acid composition of raw and hot smoked sturgeon (*Huso huso*, L. 1758). Int. J. Food Sci. Nutr..

[67] Alipour H.J., Shabanpoor B., Shabani A., Mahoonak A.S. (2010). Effects of cooking methods on physico-chemical and nutritional properties of Persian sturgeon *Acipenser persicus* fillet. Int. Aquat. Res..

[68] Kenari A.A., Regenstein J.M., Hosseini S.V., Rezaei M., Tahergorabi R., Nazari R.M., Mogaddasi M., Kaboli S.A. (2009). Amino Acid and Fatty Acid Composition of Cultured Beluga (*Huso huso*) of Different Ages. J. Aquat. Food Prod..

[69] Badiani A., Anfossi P., Fiorentini L., Gatta P.P., Manfredini M., Nanni N., Stipa S., Tolomelli B. (1996). Nutritional composition of cultured sturgeon (*Acipenser* spp.). J. Food Compos. Anal..

[70] Ghomi M.R., Nikoo M., Babaei Z. (2011). Fatty acid composition in farmed great sturgeon *Huso huso*. Comp. Clinic. Pathol..

[71] Nieminen P., Westenius E., Halonen T., Mustonen A.M. (2014). Fatty acid composition in tissues of the farmed Siberian sturgeon (*Acipenser baerii*). Food Chem..

[72] Vaccaro A.M., Bua G., Messina C.M., Santulli A., Mazzola A. (2005). Fatty acid composition of a cultured sturgeon hybrid (*Acipenser naccarii* x *A. baerii*). Food Chem..

[73] Ezejiofor T.I.N., Enebaku U.E., Ogueke C. (2014). Waste to wealth-value recovery from agro-food processing wastes using biotechnology: A review. Br. Biotechnol. J..

[74] Kundam D.N., Acham I.O., Girgih A.T. (2019). Bioactive compounds in fish and their health benefits. Asian Food Sci. J..

[75] Zilia F., Bacenetti J., Sugni M., Matarazzo A., Orsi L. (2021). From waste to product: Circular economy applications from sea urchin. Sustainability.

[76] Villamil O., Vaquiro H., Solanilla J.F. (2017). Fish viscera protein hydrolysates: Production, potential applications and functional and bioactive properties. Food Chem..

[77] Sarmadi B.H., Ismail A. (2010). Antioxidative peptides from food proteins: A review. Peptides.

[78] He S., Franco C., Zhang W. (2013). Functions, applications and production of protein hydrolysates from fish processing co-products (FPCP). Int. Food Res..

[79] Nolsøe H., Undeland I. (2009). The acid and alkaline solubilization process for the isolation of muscle proteins: State of the art. Food Bioproc. Tec..

[80] Pasupuleti V.K., Braun S., Pasupuleti V., Demain A. (2010). State of the art manufacturing of protein hydrolysates. Protein Hydrolysates in Biotechnology.

[81] Bhaskar N., Benila T., Radha C., Lalitha R.G. (2008). Optimization of enzymatic hydrolysis of visceral waste proteins of Catla (*Catla catla*) for preparing protein hydrolysate using a commercial protease. Bioresour. Technol..

[82] Klompong V., Benjakul S., Kantachote D., Shahidi F. (2007). Antioxidative activity and functional properties of protein hydrolysate of yellow striptrevally (*Selaroides leptolepis*) as influenced by the degree of hydrolysis and enzyme type. Food Chem..

[83] Ryan J.T., Ross R.P., Bolton D., Fitzgerald G.F., Stanton C. (2011). Bioactive peptides from muscle sources: Meat and fish. Nutrients.

[84] Aleman A., Gomez-Guillen M.C., Montero P. (2013). Identification of ACE inhibitory peptides from squid skin collagen after *in vitro* gastrointestinal digestion. Int. Food Res..

[85] Ovissipour M., Abedian A., Motamedzadegan A., Rasco B., Safari R., Shahiri H. (2009). The effect of enzymatic hydrolysis time and temperature on the properties of protein hydrolysates from Persian sturgeon (*Acipenser persicus*) viscera. Food Chem..

[86] Ovissipour M., Safari R., Motamedzadegan A., Shabanpour B. (2012). Chemical and Biochemical Hydrolysis of Persian Sturgeon (*Acipenser persicus*) Visceral Protein. Food Bioproc. Tec..

[87] Caldeira M., Barreto C., Pestana P., Cardoso M.A.T. (2018). Fish residue valorization by the production of value- added compounds towards a sustainable zero waste industry: A critical review. J. Sci. Eng. Res..

[88] Al Khawli F., Martí-Quijal F.J., Ferrer E., Ruiz M.J., Berrada H., Gavahian M., Barba F.J., de la Fuente B. (2020). Aquaculture and its by-products as a source of nutrients and bioactive compounds. Adv. Food Nutr. Res..

[89] Silva T.H., Moreira-Silva J., Marques A.L.P., Domingues A., Bayon Y., Reis R.L. (2014). Marine origin collagens and its potential applications. Mar. Drugs.

[90] Xie Q., Liu Y. (2022). Comparative analysis of the nutrient components in the muscle and skin tissues of hybrid sturgeon (*Acipenser baerii* ♀ *× Acipenser schrenckii* ♂) of different sizes. Aquac. Res..

[91] Bruno S.F., Ekorong F.J., Karkal S.S., Cathrine M.S.B., Kudre T.G. (2019). Green and innovative techniques for recovery of valuable compounds from seafood by-products and discards: A review. Trends Food Sci. Technol..

[92] Webb G.P., Berginc K., Kreft S. (2015). Vitamins/minerals as dietary supplements: A review of clinical studies. Dietary Supplements: Safety, Efficacy and Quality.

[93] Huang H., Wei Y., Li L., Yang X., Chen J., Pan C., Hao S. (2021). The impact of seasonal changes on the nutritional composition of hybrid sturgeon meat. Food Ind. Sci. Technol..

[94] Menon V.V., Lele S.S., Kim S.K. (2015). Nutraceuticals and bioactive compounds from seafood processing waste. Springer Handbook of Marine Biotechnology.

[95] Place E.S., Evans N.D., Stevens M.M. (2009). Complexity in biomaterials for tissue engineering. Nat. Mater..

[96] Karimzadeh K. (2018). Antihypertensive and anticoagulant properties of glycosaminoglycans extracted from the sturgeon (*Acipenser persicus*) cartilage. Curr. Issues Pharm. Med. Sci..

[97] Zheng P., Teng F., Hu M., Li Q., Xu N., Li W., Wang J., Zhang H. (2021). Optimization of the extraction process of type II collagen from sturgeon cartilage. J. Food Saf. Qual. Inspect..

[98] Zhu L., Li J., Wang Y., Sun X., Li B., Poungchawanwong S., Hou H. (2020). Structural feature and self-assembly properties of type II collagens from the cartilages of skate and sturgeon. Food Chem..

[99] Meng D., Li W., Leng X., Takagi Y., Dai Z., Du H., Wei Q. (2023). Extraction of chondroitin sulphate and type II collagen from sturgeon (*Acipenser gueldenstaedtii*) notochord and characterization of their hybrid fibrils. Process Biochem..

[100] Meng D., Wei Q., Takagi Y., Dai Z., Zhang Y. (2023). Structural Properties and Biological Activities of Collagens from Four Main Processing By-Products (Skin, Fin, Cartilage, Notochord) of Sturgeon (*Acipenser gueldenstaedtii*). Waste Biomass Valor..

[101] Li Z., Bai X., Fan Y., Jia Q., Zhang H., Hou H. (2022). Structure of type II collagen from sturgeon cartilage and its effect on adjuvant-induced rheumatoid arthritis in rats. Food Funct..

[102] Sheng Y., Qiu Y.T., Wang Y.M., Chi C.F., Wang B. (2022). Novel Antioxidant Collagen Peptides of Siberian Sturgeon (Acipenser baerii) Cartilages: The Preparation, Characterization, and Cytoprotection of H_2_O_2_-Damaged Human Umbilical Vein Endothelial Cells (HUVECs). Mar. Drugs.

[103] Luyuan L., Shengfan W., Yougui Z., Yinjun Z., Zhao W., Jianyong Z. (2019). Enzymatic preparation of sturgeon skin collagen polypeptide and its antioxidant activity. Food Ferment Ind..

[104] Atef M., Ojagh S.M., Latifi A.M., Esmaeili M., Udenigwe C.C. (2020). Biochemical and structural characterization of sturgeon fish skin collagen (*Huso huso*). J. Food Biochem..

[105] Ahmad T., Ismail A., Ahmad S.A., Khalil K.A., Kumar Y., Adeyemi K.D., Sazili A.Q. (2017). Recent advances on the role of process variables affecting gelatin yield and characteristics with special reference to enzymatic extraction: A review. Food Hydrocoll..

[106] Nikoo M., Benjakul S., Ehsani A., Li J., Wu F., Yang N., Xu B., Jin Z., Xu X. (2014). Antioxidant and cryoprotective effects of a tetrapeptide isolated from Amur sturgeon skin gelatin. J. Funct. Foods.

[107] Islam M.R., Yuhi T., Ura K., Takagi Y. (2020). Optimization of Extraction of Gelatin from the Head of Kalamtra Sturgeon (*Huso dauricus × Acipenser scherenkii × Acipenser transmontanus*). Appl. Sci..

[108] Yang Z., Zhang K., Liu Y., Wang Y., Huang W. (2021). Comparison of Physicochemical Properties of Pepsin-Soluble Collagens from Swim Bladders of Sturgeon (*Acipenser schrenckii*) and Grass Carp (*Ctenopharyngodon idella*). Sci. Technol. Food Ind..

[109] Zu X.Y., Liu W.B., Xiong G.Q., Liao T., Li H.L. (2023). Isolation, Identification, and Biological Activity Analysis of Swim Bladder Polypeptides from *Acipenser schrencki*. Foods.

[110] Hao S., Wei Y., Li L., Yang X., Cen J., Huang H., Lin W., Yuan X. (2015). The effects of different extraction methods on composition and storage stability of sturgeon oil. Food Chem..

[111] Wu L.B., He R.Y., Yu H., Wang H.L., Liu Z.G. (2022). Enzymatic extraction of sturgeon fish oil and its intervention on non-alcoholic fatty liver disease. J. Food Saf. Qual..

[112] Lee H.S., Lee Y.K., Park J.H., Kim S.H., Park C.S., Kim K., Lee C. (2024). Therapeutic efficacy and mechanism of solubilized sturgeon oil in a mouse model of house dust mite-induced atopic dermatitis. J. Func. Foods.

[113] Qiang H., Mengcheng R., Hualin W., Rulong C., Zhiguo L. (2017). Effect of sturgeon oil on intestinal flora balance in mice with high-fat induced nonalcoholic fatty liver disease. J. Wuhan Univ. Light. Ind..

